# Tools advancing the detection of cell surface RNAs

**DOI:** 10.1093/procel/pwaf089

**Published:** 2025-11-03

**Authors:** Ryan A Flynn

**Affiliations:** Stem Cell and Regenerative Biology Program, Division of Hematology/Oncology, Department of Pediatrics, Boston Children’s Hospital, Boston, MA, 02115, United States; Department of Stem Cell and Regenerative Biology, Harvard University, Cambridge, MA, 02138, United States; Harvard Stem Cell Institute, Harvard University, Cambridge, MA, 02138, United States

While the cell surface has long been studied in the context of its critical cell biology roles for mechanisms that relate to proteins, lipids, and glycans, only recently has RNA become an active player. Classically, surface-presented biopolymers such as proteins and lipids are glycosylated, facilitating their trafficking, folding, and biological activity in the extracellular space. The discovery that small noncoding RNAs also serve as templates for N-glycosylation (glycuronans (Flynn et al. 2019; 2021)) and the later identification of a covalent linkage between these two biopolymers (Xie et al. 2024) provided a framework to consider a broader suite of cell surface RNA biology. 

The ability to visualize cell surface RNA biology can help to better understand its mechanistic features. Most technologies enabling imaging require knowledge of what to image; therefore, sequencing of surface-presented RNAs is an important first step. Glycans on RNAs were found to be linked to small noncoding RNAs, including tRNA, Y RNA, small nuclear RNAs (snRNAs), and small nucleolar RNAs (snoRNAs) (Flynn et al. 2019; 2021), and biochemical isolation of membranes coupled to sequencing also provided evidence that certain longer RNAs are also associated to membrane (Huang et al. 2020). Since these studies, significant efforts have been put forward to image glycoRNAs (Ma et al. 2024; Liu et al. 2024; Ren et al. 2025), many of which leverage proximity between glycan- and RNA-targeting reagents for specificity. The ability to more broadly profile cell surface RNAs with sequencing has lagged relatively behind these proximity-based tools.

In an effort to address the above, Jiang et al. (2025) have focused on developing two related methods named Intact-Surface-FISH and in-situ Amplification of Membrane Outer-surface RNAs (AMOUR) that are read out by imaging and sequencing, respectively.

Intact-Surface-FISH was developed to directly visualize RNAs exposed on the extracellular side of the plasma membrane. In this assay, live cells were incubated with fluorescently labeled DNA probes complementary to candidate surface RNAs. To retain surface specificity, no fixation or permeabilization was performed; instead, gentle washing with 1 mmol/L ATP replaced formamide to maintain cell viability and prevent denaturation. This was initially applied to cells using a random 20-mer DNA probe, which produced membrane-localized signals on HeLa and HEK293T cells that were strongly reduced after RNase treatment, confirming that the detected signal was RNA-dependent.

Extending this principle to a discovery strategy, the authors developed the AMOUR method. Here, fixed, intact cells were immobilized on concanavalin-A–coated magnetic beads to enable experimental manipulation of whole cells. Next, DNA probes containing a T7 promoter and random 9-mer overhang were added to hybridize to surface RNAs. T7 RNA polymerase then performed in-situ transcription, “jumping” from the probe onto surface RNA templates to generate amplified RNA products for reverse transcription and eventually read out using high-throughput sequencing. While certain methodological changes were made between the FISH and AMOUR protocols, specific efforts were made to demonstrate that the signal generated from AMOUR experiments represents cell surface RNAs, including RNase pre-treatment of live cells, comparison to lysed-cell controls, and membrane quencher exclusion.

Using a combination of AMOUR data and Intact-Surface-FISH, numerous types of RNAs were characterized to be on the cell surface, including tRNAs (especially mitochondrial tRNAs), snoRNAs, snRNAs, Y RNAs, and miscellaneous non-coding RNAs; largely aligning with the classes of RNAs previously discovered to by glycoRNAs (Flynn et al. 2021; Zhang et al. 2024). Certain longer noncoding RNAs, such as XIST, important for X-chromosome silencing and associated with autoimmunity (Loda and Heard 2019; Dou et al. 2024; Yan et al. 2025) as well as mitochondrial rRNA (e.g., MTRNR2) were found in the AMOUR data, with biological context for these observations to follow.

These data point to a complex landscape of RNA on the cell surface, yet many questions remain to better contextualize this biology. Sequencing-based methods like AMOUR that can sample rare cell populations will allow new maps of cell surface RNAs to be defined. Validating and contextualizing data generated from these sequencing profiles with techniques like Intact-Surface-FISH will be important. Developing ways to obtain higher resolution views as well as multi-target information will additionally deepen our understanding of the organization of RNA on the cell surface. Further, it will be of significant interest to understand the relationship between any RNA detected on the cell surface and its chemical modification landscape, with a particular focus on ­glycans, considering both the type and sites of modifications.

## Conflict of interest

None declared.
